# Supplementary material for: An exploratory assessment of the impact of a novel risk assessment test on breast cancer clinic waiting times and workflow: a discrete event simulation model
Source: BMC Health Serv Res. 2022 Oct 29;22:1301. doi: 10.1186/s12913-022-08665-0 (PMC9617530; doi:10.1186/s12913-022-08665-0)
Supplement: Supplementary file 4 — Supplementary Material 4 [file 12913_2022_8665_MOESM4_ESM.docx]

**Strengthening the Reporting of Empirical Simulation Studies (STRESS)**

**Discrete-event simulation guidelines STRESS-DES**

| **Section/Subsection** | **Item** | **Details/ location in Manuscript where details can be found** | |
| --- | --- | --- | --- |
| 1. **Objectives** |  |  | |
| Purpose of the model | 1.1 | In collaboration with the Leeds Teaching Hospitals NHS Trust (LTHT) and the University of Leeds, PinPoint Data Science Ltd have developed a risk assessment tool, which is designed to determine patients’ risk of breast cancer based on a number of routine blood tests (including haematological, biochemical and tumour markers). These individual tests are combined within an algorithm (the “PinPoint test”) to provide a calibrated risk probability of cancer (a score between zero and one, with higher values indicating higher risk).  The aim of this early exploratory evaluation was to assess the potential impact of the PinPoint test (PinPoint Data Science Ltd) on the flow of patients through secondary care breast cancer clinic services in the UK National Health Service (NHS), focusing on a specific case study of the Leeds Teaching Hospitals Trust (LTHT) breast cancer clinic.  This evaluation focuses on the potential use of PinPoint as a rule-out test for patients with very low risk of cancer (i.e. avoiding secondary care referrals in this group). | |
| Model Outputs | 1.2 | Two primary outcomes were evaluated: (i) the percentage of patients seen in the breast cancer clinic in under two weeks (i.e. meeting the two week wait (TWW) referral target), and (ii) the number of ‘overspill’ appointments generated (i.e. where patients have to return to the clinic for further diagnostics due to insufficient same-day clinic capacity). Together these outcomes represent how well the referral system achieves timely diagnoses for patients within an efficiently functioning system. | |
| Experimentation Aims | 1.3 | The experimental aims of this evaluation were to determine the potential impact of the PinPoint test on the ability of secondary care breast cancer clinics to see patients in a timely and efficient manner, in order to determine if there is utility in pursuing further research on the PinPoint test.  Due to current uncertainty around the impact of the PinPoint test (in particular relating to expected clinical management and outcomes for the ~20% of patients initially ruled out by the PinPoint test), the findings from this analysis should be considered as exploratory only. | |
| 1. **Logic** |  |  | |
| Base model overview diagram | 2.1 | The model structure is illustrated in **Figure 1** of the manuscript.  The model is intended to reflect the flow of patients through the LTHT breast cancer clinic: a medium-to-large sized clinic which sees around 10,500 patients along the TWW pathway annually (based on 2019 clinic audit data). The model tracks individual patients from their initial general practitioner (GP) presentation, through to clinic services (including initial assessment, mammogram, ultrasound and biopsy), accounting for available clinic resources (staff and clinic rooms/imaging devices). Patients exit the model with a final diagnosis of breast cancer (following a multi-disciplinary team [MDT] meeting) or no breast cancer.  In the standard care arm, all patients receive a referral to the clinic at their initial GP appointment; whilst in the PinPoint arm, referrals are based on whether patients receive a low- or high-risk PinPoint test result. Depending on the number of patients already in the queue for the clinic, referred patients have to wait for a period of time for the next available slot; similarly once in the clinic, patients must wait for the required staff and rooms to be available in order to undergo their required investigations. | |
| Base model logic | 2.2 | This section provides an overview of the model logic. In conjunction with this, please refer to **Additional File 1** of the manuscript to see a list of the model clock properties and input parameters.  Patients with suspected breast cancer enter the model. A unique identifier label is assigned to all new arrivals at this point, to enable individual entities in the model to be tracked for internal validity checks. In addition, for all new patients entering the model, the model simulation time is compared to the model ‘warm up’ duration (12 weeks), and if the simulation time is greater than the warm up duration, a global variable counting the number of patients arriving into the model is increased by +1. This ensures that a count is kept of the number of patients entering the model during the results collection (i.e. evaluation) period.  At the ‘GP’ activity, a ‘time stamp’ label is set, which records each patient’s time of GP entry in the model. This is used to calculate each individual’s time to secondary care, later in the model. A label for each patient’s breast cancer status is also assigned, according to the breast cancer incidence variable (see **Additional File 1**). No resources or timings are applied at the ‘GP’ activity (i.e. the GP is assumed to have infinite capacity; the focus of this model is on evaluating patient workflow within the secondary care clinic).  Patients enter the queue for the ‘TWW Referral’ activity, and are assigned a minimum wait time of 1 week.  For the ‘TWW Referral’ activity, prior to a morning (AM) or afternoon (PM) clinic opening (see section **2.5.3 Resources** for details on the weekly clinics), the ‘Enter Breast Cancer Clinic’ activity collects the next set of patients to enter the upcoming clinic (i.e. to move into the queue for ‘Initial assessment’). Ten minutes prior to each AM or PM clinic, dummy ‘clinic opening’ shifts are set to open for a brief period (1 minute). Logic code at the ‘Enter Breast Cancer Clinic’ activity (“Routing In” logic code) checks if any of the dummy clinic opening shifts are active, and if so, further logic code is run to determine the number of patients to be collected by the ‘Enter Breast Cancer Clinic’ activity and placed in the queue for the clinic ‘Initial Assessment’ activity (this number is based on the number of patients in the queue for the clinic – see section **2.4 Algorithms** for details).  On entry to the clinic (i.e. on moving out of the ‘Enter Breast Cancer Clinic’ activity and into the queue for ‘Initial assessment’), a time stamp label is set to record each patient’s time of entering the clinic. This is used to calculate each patient’s time to clinic (assigned as another label), based on subtracting their time of GP entry from the time of clinic entry.  For each of the clinic activities, shift dependant resources are assigned (section **2.5.3 Resources** for details). Only when required resources for an activity are available (i.e. on shift, and not already working on an activity) can the next patient in the queue be picked up and moved into the associated activity.  At the ‘Initial assessment’ activity, the proportion of patients without breast cancer who are referred for imaging (vs. immediately discharged) is set, based on drawing from an assigned distribution for this proportion (see **Additional File 1**). Those patients immediately discharged are routed out of the clinic and enter the ‘Exit clinic’ activity. Of those patients who go on to receive further investigations (including all patients with breast cancer and a proportion of those without breast cancer), each patient is assigned a clinic pathway label (a number, from 1 to 9, indicating the sequence of investigations that patient is required to undergo at the clinic). The proportion of patients following each of the nine possible clinic pathways is based on LTHT PLICs data for 2018/19 (see **Additional File 1** and section **3.1 Data sources** for more details). Patients are then routed out from the ‘Initial assessment’ activity to the next appropriate clinic activity, based on their assigned pathway label value.  Once a clinic closes, any patients remaining in any of the queues for clinic activities are routed out to the ‘Delayed clinic assessments’ activity. The clinic activity queue from which patients are collected at this point is recorded within a label, to enable patients to be returned to the correct queue upon their return into the clinic. Patients are assumed to have to wait at least 3 hours before re-entering the clinic to complete their necessary sequence of investigations (i.e. they must wait at least 1 clinic before re-entering). After 3 hours, patients pass through the ‘Clinic Rebooking’ activity and enter the queue for ‘Clinic returns’. Similar to the process applied at the ‘Enter Breast Cancer Clinic’ activity, dummy ‘clinic opening’ shifts are also utilised to set a brief period of time prior to an AM or PM clinic opening, wherein the number of clinic returns to be included in the next clinic (i.e. picked up by the ‘Clinic returns’ activity and placed back in the appropriate clinic activity queue) is determined. Twenty minutes prior to each AM or PM clinic opening, dummy ‘clinic opening’ shifts are set to open for a brief period (1 minute). Logic code at the ‘Clinic returns’ activity (“Routing In” logic code) checks if any of the relevant dummy clinic opening shifts are active, and if so, further logic code is run to determine the number of returning patients (or ‘overspills’) to be collected by the ‘Clinic returns’ activity (a maximum number of 5 overspills are collected for each of the full clinics, and 6 for the Tuesday AM add-on clinic). Each patient is routed out to the clinic activity queue they were previously waiting in before being sent to the ‘Delayed clinic assessments’ activity, based on the recorded label mentioned previously. Setting the dummy ‘clinic opening’ shifts for overspill returns to occur before to the dummy ‘clinic opening’ shifts for new patients, means that overspill patients are placed at the top of the clinic activity queues (and therefore seen before new patients).  Once patients have completed their required sequence of activities in the clinic, they are routed out either to the ‘Exit clinic’ activity (for all patients without breast cancer who do not undergo biopsy), or to the ‘Exit clinic: MDT review’ activity (for patients who undergo a biopsy – including all patients with breast cancer, and a proportion of patients without breast cancer). Those patients undergoing the MDT review activity are routed out either to the ‘Breast cancer detected’ activity, or the ‘Breast cancer not detected’ activity, depending on their true disease status label. At these two end-point activities, logic code is applied to calculate and store individual and global results (e.g. logging whether or not patients reached the clinic within the TWW target; counting the number of patients reaching the different model end-points; and counting the number of clinic overspill cases). | |
| Scenario logic | 2.3 | In the PinPoint arm of the model, all patients seeing the GP are given the test, and enter a separate ‘Test’ activity. Logic is assigned at the ‘Test’ activity to determine, based on each individual’s true disease status and the diagnostic sensitivity and specificity of the test, what result is received (i.e. ‘low’ or ‘high’ risk for cancer). The PinPoint test result is recorded as a label for each individual, and all those patients with a high risk result are routed out to a ‘High risk’ activity; and all those with a low risk result are routed out to a ‘Low risk’ activity.  Patients receiving a high risk result are referred to secondary care, and enter the queue for the ‘TWW Referral’ activity (from the ‘High risk’ activity) and subsequently follow the same pathways and logic as outlined in section 2.2 above for the standard care arm.  Patients receiving a low risk result are not initially referred to secondary care. Based on the current uncertainty around what may happen to patients receiving a low risk PinPoint result, two primary scenarios for the PinPoint strategy were considered in the experimental analysis:   - Scenario 1: GPs only refer patients with high-risk results (i.e. 100% adherence to testing), and all patients with cancer who receive a low-risk test result are assumed to return after 6 weeks with persisting symptoms and receive a delayed clinic referral. - Scenario 2: GPs randomly overrule 20% of low-risk results (cancerous and non-cancerous cases), and a further 10% of patients without breast cancer with a low-risk test result are also assumed to return and receive a delayed referral at six weeks.   Under scenario 1, at the ‘Low risk’ activity point, all patients without breast cancer exit the model. All patients with breast cancer enter a queue for a ‘Delayed referrals’ activity, and are assumed to return after 6 weeks (i.e. a minimum queue time of 6 weeks is applied at the queue for ‘Delayed referrals’). From the ‘Delayed referrals’ activity, patients then enter the queue for ‘TWW Referrals’ and subsequently follow the same pathways and logic as outlined in section 2.2 above.  Under scenario 2, an additional activity of ‘GP override’ following the ‘Low risk’ result activity is also included. Logic code is applied at the ‘Low Risk’ activity point to first randomly route out 20% of patients to the ‘GP override’ activity, from which individuals are routed to the queue for ‘TWW Referral’. Further code is then applied to set a 10% probability for patients without breast cancer entering the ‘Delayed referrals’ queue. The remaining patients without breast cancer exit the model (as in scenario 1), and all patients with breast cancer enter the queue for ‘Delayed referrals’ (as in scenario 1).  In addition to the PinPoint scenarios outlined above, a range of deterministic sensitivity analyses were run to explore the impact of individual parameters on the model results (see **Additional File 3** for the sensitivity analysis results). These analyses were undertaken by altering the parameter of interest in the model and re-running the analysis.  Further service reconfiguration scenarios were also explored based on: (1) removing the Tues AM add-on clinic; (2) removing a full clinic (arbitrarily chosen as Wed AM); and (3) removing the Tues AM and Wed AM clinics. These options were applied together with the Pinpoint Scenario 2 parameters. | |
| Algorithms | 2.4 | Process for assigning the number of new patients seen in clinic:  The default number of new patients booked into each clinic was set to 25 for full clinics, and 10 for the Tues AM add-on clinic. For the Tues AM clinic a maximum of 10 new patients could be included in each clinic; whilst for the full clinics, up to 34 patients could be included depending on the total number of patients currently in the referral queue.  Within the ‘Enter Breast Cancer Clinic’ activity, logic code is used to first check if a full or add-on clinic is due to open. If the Tues AM add-on clinic is due to open, then the number of patients to be collected for the clinic is set by checking the number of patients in the queue for the ‘Enter Breast Cancer Clinic’ activity: if this queue is greater than or equal to 10, then the default maximum clinic number of 10 patients is collected; if the queue is less than 10, then the collection number is set equal to the number of patients in the queue.  If a full AM or PM clinic is due to open, then the total number of patients queueing within the queue for the ‘TWW Referral’ activity and the ‘Enter Breast Cancer Clinic’ activity is checked. If the total number queuing for the clinic is greater than 500, then the number to be collected for the clinic is set to the upper maximum value of 34. If the total number queuing is less than or equal to 500, then iterative logic is applied to determine which number of patients should be collected (using the numbers reported in **Additional File 1**), down to the default collection number of 25 for a queue size of 380 or less. Finally, if the queue size for the ‘Enter Breast Cancer Clinic’ is less than 25, then the collection number is set equal to the size of the queue for the ‘Enter Breast Cancer Clinic’ activity. This process ensures that the clinic numbers reflect a degree of ‘responsiveness’ to the queue, in line with real-life practice (see main manuscript for further discussion on this). | |
| Components | 2.5 | 2.5.1 Entities | The model entities consist of patients attending their GP with symptoms of breast cancer, who meet the National Health Service (NHS) two week (urgent) referral criteria. |
|  |  | 2.5.2 Activities | The key model activities are illustrated in **Figure 1** of the manuscript.  The only clinic activity not illustrated in **Figure 1** is that of two sub-processes used for the ‘Mammogram’ and ‘Ultrasound’ activities. Both of these imaging activities are assumed to require and initial ‘patient preparation’ activity, in which a Grade 2 or 6 member of staff (see section **2.5.3 Resources** details) escorts the patient to their imaging room, and prepares them for the imaging activity. Both the mammogram and ultrasound imaging activities do not require either a Grade 2 or 6 member of staff to be undertaken, and hence the initial patient preparation activity is modelled as a separate activity with separate resource requirements, but is grouped with the associated subsequent imaging activity using Simul8’s ‘sub-process’ feature. This means that a new patient is only allowed into the patient preparation activity if the grouped imaging activity is free (i.e. no patients are currently being processed in the grouped imaging activity). Note that the biopsy activity also requires a patient preparation step, however biopsy already requires a Grade 2 or Grade 6 staff member to be undertaken (see section **2.5.3 Resources** detail), and therefore for simplicity the patient preparation step for that activity was assumed to be captured within the biopsy activity itself. |
|  |  | 2.5.3 Resources | The clinic activities modelled (i.e. ‘Initial assessment’, ‘Mammogram’, ‘Ultrasound’ and ‘Biopsy’) are only ‘open’ (i.e. able to accept new patients) when one of 8 weekly clinics is underway. In line with the LTHT clinic schedule (as of 2019), the model includes seven weekly ‘full’ clinics (Mon AM, Tues PM, Weds AM & PM, Thurs AM & PM, and Fri AM), and one weekly ‘add-on’ clinic with a reduced capacity (Tues AM; this clinic was first introduced in early 2018 as a temporary measure to alleviate pressure on the clinic, but has since been permanently adopted due to persisting demand). All clinics last four hours (AM = 09:00 to 13:00; PM = 14:00 to 18:00), with up to fifteen minutes of staff overtime allowed to complete activities if necessary.  The clinic staff includes surgical staff (consultants, nurse practitioners [NPs] and physician associates [PAs]); and radiographer staff (Band 6 sonographers and non-sonographers, Band 7 sonographers and Grade 2 assistants). Staff numbers working during each clinic were set to match the LTHT staff schedule, and staff availability within shifts was set based on expert opinion as to the amount of time each staff member would typically spend on ‘other’ activities (e.g. administrative tasks and follow-up appointments) (see **Additional Files 1 and 2**). Staff availabilities were set using Simul8’s automatic ‘Availability’ function (i.e. no set values for the frequency or length of absences for staff were assigned).  Specific staff are required for each of the clinic activities. Initial assessment requires a member of surgical staff (consultant, NP, or PA); mammogram requires a qualified radiographer (Band 6 sonographer/ non-sonographer, or Band 7 sonographer); ultrasound requires a sonographer (Band 6 or 7); and Biopsy requires a consultant and either a grade 2 or any grade 6 member of staff. In addition, each of the imaging and biopsy activities requires an initial ‘patient preparation’ step, wherein a Grade 2 or Grade 6 member of staff escorts the patient to their required room and prepares them for the procedure to be undertaken. Note that, whilst specific staff are required for clinic activities, patients are not assigned to a specific member of staff – rather, as soon as any relevant staff member becomes available for the patient’s next required activity, the patient can undergo that activity.  The maximum number of each clinic activity able to be undertaken at any given time was based on the number of rooms (each with one available imaging device) available at the LTHT clinic (four initial assessment; three mammogram; four ultrasound; and four biopsies). The actual number of each activity undertaken at any point depends also on the availability of staff: an activity can only go ahead if both a room and required staff are available. The median times taken to complete each activity was based on expert opinion (initial assessment = 10 minutes; mammogram = 20 minutes; ultrasound = 20 minutes; biopsy = 30 or 45 minutes [each with 50% chance of occurring]), allowing for some variation around the expected timings (see **Additional** **File** **1**). |
|  |  | 2.5.4 Queues | All queues in the model follow a first in first out discipline. |
|  |  | 2.5.5 Entry/Exit Points | Arrival and exit points of the model are illustrated in **Figure 1** of the manuscript.  All patients enter the model at the ‘Suspected BC [breast cancer] GP Arrivals’ point (illustrated as a green arrow to the left of the ‘GP’ activity in **Figure 1**). In the standard care arm, all patients without breast cancer exit the model at the ‘Referrals No BC Exit’; whilst patients with breast cancer exit the model at the ‘Referrals BC Exit’ point. In addition, in the PinPoint arm of the model, patients without breast cancer who receive a low risk test result, may exit the model at a ‘Non referrals No BC Exit’ point (the proportion exiting at this point depends on the assumptions applied around the proportion of GP adherence to test results, and the proportion of patients without breast cancer returning with persisting symptoms and receiving a delayed clinic referral).  Updating of global model results variables (e.g. the number of patients meeting the TWW target) is undertaken at the two primary end activities: ‘Breast cancer not detected’ and ‘Breast cancer detected’. |
| 1. **Data** |  |  | |
| Data sources | 3.1 | A list of model input parameter values alongside associated data sources is provided in **Additional File 1**.  Two key sources of data were utilised for this analysis:   - LTHT Leeds PLICs data: this consisted of a bespoke data extract from the LTHT electronic health record and associated Leeds Patient Level Information and Costing System (PLICS) dataset for 2018/19. This data was used to inform: the breast cancer incidence rate and the sequence of activities undertaken by patients within the LTHT breast cancer clinic. - LTHT breast cancer clinic 2019 audit data: this consisted of routinely recorded data, as recorded by LTHT staff, on the number of new and overspill appointments undertaken in every LTHT clinic, how many patients received some form of imaging (rather than being directly discharged), and how many overspill appointments were produced in each clinic. | |
| Pre-processing | 3.2 | Analysis was undertaken on the LTHT clinic audit data – this consisted of simple tallying and averaging of clinic numbers, overspill appointments, and immediate discharges, to derive key parameters as listed in **Additional File 1**. | |
| Input parameters | 3.3 | A list of model input parameters are provided in **Additional File 1**. | |
| Assumptions | 3.4 | Key assumptions applied in the model are listed below:   - The arrival of patients into the model is assumed to be constant – temporal variation in patient arrivals is not considered in this model. - Limited GP capacity is not considered in the model – i.e. GPs are assumed to have infinite capacity for the purpose of this analysis. - Newly referred patients are expected to have to wait a minimum of 1 week before being booked into the next available clinic. - Returning patients (i.e. overspill appointments) are expected to have to wait at least 1 day before being booked into the next clinic. - Sudden staff absences or sick days are not considered in this model. | |
| 1. **Experimentation** |  |  | |
| Initialisation | 4.1 | A model warm up period of 12 weeks was applied, and the model runs in minutes (for the purpose of aiding understanding, minute values have been converted into weeks where appropriate in the description of the model). | |
| Run length | 4.2 | The model was run for a period of one year (52 weeks). | |
| Estimation approach | 4.3 | All model analyses were based on running 150 replications (trials) of the model, to account for first order uncertainty. All analyses used a base random number seed of 20. | |
| 1. **Implementation** |  |  | |
| Software or programming language | 5.1 | The model was constructed in Simul8 ([https://www.simul8.com](https://www.simul8.comD)). | |
| Random sampling | 5.2 | All model analyses were based on running 150 replications (trials) of the model, to account for first order uncertainty in the model. All analyses used a base random number seed of 20. | |
| Model execution | 5.3 | All queues in the model work on a first in first out basis, such that those patients who have been waiting the longest will always be selected first for activities when resources become available. | |
| System Specification | 5.4 | The model was run on a Lenevo Thinkpad Laptop X280 (intel Core i5, 8^th^ generation). Without using parallel processing, the standard care arm of the model took approximately six hours to run and the PinPoint arms of the model took approximately five hours to run. | |
| 1. **Code Access** |  |  | |
| Computer Model Sharing Statement | 6.1 | Simul8 software can be purchased via the Simul8 website: [https://www.simul8.com](https://www.simul8.comD). The model code can be requested via the corresponding author at: [a.f.c.smith@leeds.ac.uk](mailto:a.f.c.smith@leeds.ac.uk). | |
